# Eight years of antibiotic consumption at a primary care outpatient facility in Trinidad and Tobago 2011–18: a synopsis of consumption trends

**DOI:** 10.1093/jacamr/dlab162

**Published:** 2021-10-20

**Authors:** Raveed Khan, Misty Gangar, Melanie Gangar, Shastri Motilal

**Affiliations:** 1 Department of Paraclinical Sciences, The University of the West Indies, St Augustine, Trinidad and Tobago; 2 Tallahassee Memorial Healthcare, Tallahassee, FL, USA; 3 Superpharm Limited, San Juan, Trinidad and Tobago

## Abstract

**Objectives:**

To explore antibiotic consumption and surveillance patterns in Trinidad and Tobago.

**Methods:**

A retrospective observational study was conducted. Stock requisition and logbooks from a District Health Facility in Trinidad were examined for the period 2011–18. Daily Defined Doses (DDDs) for each antibiotic were computed and extrapolated to represent the antibiotic consumption per 1000 residents within the population.

**Results:**

The mean consumption across the years was 2.917 DDD per 1000 residents per day. The most consumed antibiotics were cefuroxime, amoxicillin/clavulanic acid, and azithromycin, with mean DDDs of 0.879, 0.695 and 0.373 respectively. The least consumed antibiotics were cefaclor and clarithromycin, with DDDs of 0.0006 and 0.0005, respectively.

**Conclusions:**

Trinidad and Tobago is not mentioned in the WHO surveillance report on antibiotic consumption. Our most recent (2018) estimate of total antibiotic consumption was 3.224 DDD per 1000 habitants per day. This figure is an underestimate, as data was derived solely from the public sector. Notwithstanding, this data is novel and can provide a baseline for future comparison and development of national surveillance programmes.

## Introduction

The Global Action Plan (GAP) on antimicrobial resistance was adopted by member states at the World Health Assembly in 2015. One of the objectives of the GAP is to optimize the use of antimicrobial medicines, thereby underscoring the importance of monitoring the consumption of antimicrobials. Data on the consumption of antimicrobials have several uses, including exploring the relationships between exposure to antimicrobials and the development of antimicrobial resistance (AMR).^1^

The emergence and dissemination of antibiotic resistance is now understood to be an unavoidable aspect of bacterial evolution following the consumption of antibiotics.^2^ This relationship between the occurrence of resistance and the consumption of antibiotics has been subsequently reported.^3^^,^^4^

Resistant bacteria are expected outcompete other microbial communities in a context where antibiotics are administered at relatively high levels, which means that local concentrations are well above the MICs. This awareness has led to series of reports and recommendations that intend to educate and improve practices of health professionals and consumers, in order to preserve the effectiveness of our therapeutic armamentarium.^5–8^

Recently a Caribbean-wide point prevalence survey showed high usage of β-lactam antibiotics, especially third-generation cephalosporins, as well as quinolones, macrolides and a considerable degree of carbapenem usage.^9^

In Trinidad and Tobago, extensive and inappropriate use of third-generation cephalosporins has been documented in a tertiary care setting.^10^ However, there is a paucity of data on antimicrobial consumption in the primary care setting.

An appreciation of the need to have this usage quantified can be gleaned by understanding the culture and practice of the local pharmacies within Trinidad and Tobago. The service is divided between the public and private sectors. There are public hospital outpatient pharmacies, which are funded by the government, and private pharmaceutical companies. The country’s Food and Drugs Act classifies antibiotics as controlled drugs to be dispensed by prescription only. An anomaly in the law is its strict application only to those drugs defined by the term ‘antibiotics’ *per se* and not to all antimicrobials. Therefore, agents such as co-trimoxazole and the quinolones, which do not fall under this regulation, are available without prescription. It is widely known that antimicrobial agents can be obtained at pharmacies without a prescription, and pharmacists simply dispense these drugs as over-the-counter medications in response to requests from customers.^11^

This report is novel as it establishes antibiotic consumption patterns within a community setting in Trinidad and Tobago. Trinidad and Tobago’s last population count was 1 324 699 people (2011 census). The community of Chaguanas where this study was conducted has a population of 84 216 and this number was used in computing consumption rates.^12^

## Methods

Data was collected from the stock requisition and logbooks at a primary care-based outpatient public sector District Health Facility in Trinidad, namely the Chaguanas District Health Facility. This was done in the months of January to June 2019.

Stock requisition and logbooks for the years 2011–18 were examined. Antibiotic consumption data was computed using the ATC classification system and Defined Daily Dose (DDD) methodology that was developed by the WHO.^13^ Topical antibiotics were not included due to unavailability of ATC codes for those prescribed.

Data was extrapolated to represent the antibiotic consumption per 1000 residents within the study population per day (DID) to standardize the reporting metric for comparison with other estimates. Data was subsequently inputted into Statistical Package for the Social Sciences Version 23 for analysis.

Ethics approval was obtained from the University of the West Indies and the North Central Regional Health Authority with jurisdiction over the operations of this facility.

## Results

**Figure 1.**
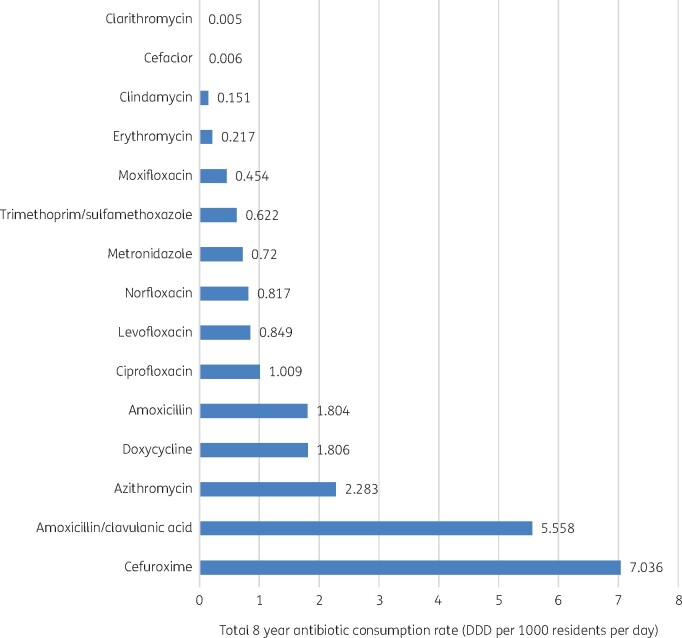
Mean antibiotic consumption rates (Public Sector only).

Analysis of the mean DDDs revealed that the most-consumed antibiotics were cefuroxime, amoxicillin/clavulanic acid, and azithromycin, consecutively. The least-consumed antibiotics were clarithromycin and cefaclor (Figure 1).

**Figure 2.**
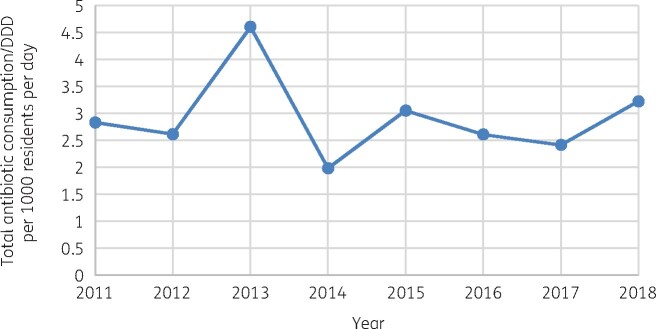
Trend of total antibiotic consumption for the years 2011–18 (Public Sector only).

The mean of total consumption across the years for all antibiotics consumed was 2.917 DDD per 1000 residents per day. The highest total consumption was in 2013, with 4.603 DDD per 1000 residents per day, and the lowest total consumption was reported in 2014, with 1.986 DDD per 1000 residents per day recorded (Figure 2).

The most-prescribed antibiotic was cefuroxime with 7.036 DDD per 1000 residents per day. Amoxicillin/clavulanic acid was the second-most-prescribed antibiotic with 5.558 DDD per 1000 residents per day. Azithromycin was the third-most-consumed antibiotic accounting for 2.283 DDD per 1000 residents per day.

## Discussion

Internationally, projects have been undertaken to collect antibiotic usage data through national surveillance systems. In the absence of a national surveillance system with a repository of such data, we have collected and analysed data over an 8 year period, thereby generating useful baseline metrics that can be extrapolated and from which meaningful conclusions can be drawn. Indeed, similar work, albeit on a larger scale, performed by the European Study on Antibiotic Consumption (ESAC) project produced a valid time series of antibiotic-use data. Those data enabled improvements to the study of determinants of use, the evaluation of governmental antibiotic consumption policies and the investigation of the associated emergence of antibiotic resistance.^14^

The computation of DDDs allows for comparison locally and internationally, thereby facilitating informed decision making as it relates to antibiotic procurement and prescribing, and provides a basis for antimicrobial resistance and stewardship programmes.

Our DDD computations came from the public sector arm of one ambulatory primary care-based facility and does not account for private sector or inpatient consumption. This therefore reduces our ability to generalize and extrapolate this metric in order to estimate national levels of antibiotic consumption. On the other hand, the fact that our data was sourced from public records suggests reasonable levels of documentation and distribution, thereby enhancing the validity and reliability of our findings.^1^

The only metric available for comparison with neighbouring countries is that for public sector only antibiotic consumption recorded by Costa Rica in 2016 of 14.18 DDDs.^15^ The corresponding figure in Trinidad and Tobago for the year 2016 was 2.61 DDDs, which is substantially lower. This can be explained by the fact that our data was derived from the Public Primary Care sector only and therefore did not include the Public Secondary and Tertiary Care sectors. In addition, the Costa Rican data was sourced from wholesalers as opposed to public sector procurement records, which was the source for our data.

In Trinidad, antibiotic prescribing is regulated by the Antibiotics Act chapter 30:02.^16^ Antibiotics dispensed at all public institutions do not incur a direct cost to the patient since this is funded by the government. However, allocations from the national budget are issued every year for the provision of healthcare services, including pharmaceuticals. The magnitude of the funding received impacts on the types and quantities of antibiotics that are procured and subsequently made available for dispensing at Public Health facilities. The unavailability of preferred antibiotics may lead to prescription of alternatives for patients who are unable to afford to purchase the preferred antibiotic. This in turn can lead to increased consumption of alternative or second-line antibiotics not accounted for in this study.

With regards to the pattern of consumption, WHO has stated that in most countries, amoxicillin and amoxicillin/clavulanic acid were the most frequently consumed antibiotics; these belong to the Access category of the Model List of Essential Medicines, which includes antibiotics recommended as first- or second-line therapy for common infectious diseases and that should be available in all countries.^15^

The finding of cefuroxime being the most-consumed antibiotic is concerning as it belongs to the Watch group of antibiotics with high resistance potential compared with those belonging to the Access category. A study from India also found cefuroxime to be amongst the most commonly prescribed antibiotics. The authors went on to state that although second- and third-generation cephalosporins offer several advantages, their use could be attributed to combination of factors such as changing prescribing practices, increasing antimicrobial resistance to other antibiotic classes and lack of availability of first-line penicillin antibiotics belonging to the Access group.^17^

Azithromycin also belongs to the Watch group of antibiotics. The second highest level of consumption of 2.13 DDDs per 1000 inhabitants per day was reported in the Austrian primary care sector in the year 2019.^18^ This suggests that it is not unusual to prescribe macrolides in primary care but, as with cephalosporins, care must be taken to reduce the development of resistance to this class of antibiotic. Indeed, research including a systematic review reported macrolide resistance after azithromycin distribution in three of five organisms studies^19^ as well as the emergence of azithromycin resistance in *Vibrio fluvialis* isolates from diarrhoeal patients in Kolkata, India.^20^

Our findings provide useful insight into the quantities and patterns of antibiotics used in our country. It is recommended that further research be undertaken involving the private sector and secondary/tertiary care settings in order to obtain a better estimate of national consumption levels.

It is envisaged that these unprecedented findings will assist in the development of a national system for antibiotic consumption and surveillance as recommended by the WHO. It is also recommended that local antimicrobial resistance patterns be explored and related to our findings in order to pioneer national antimicrobial stewardship programmes that will reduce levels of antibiotic resistance, enhance patient outcomes and ultimately provide more cost-effective care of infectious diseases.
